# Omics-Based Analytical Approaches for Assessing Chicken Species and Breeds in Food Authentication

**DOI:** 10.3390/molecules26216502

**Published:** 2021-10-28

**Authors:** Goh Dirong, Sara Nematbakhsh, Jinap Selamat, Pei Pei Chong, Lokman Hakim Idris, Noordiana Nordin, Fatchiyah Fatchiyah, Ahmad Faizal Abdull Razis

**Affiliations:** 1 Natural Medicines and Products Research Laboratory, Institute of Bioscience, Universiti Putra Malaysia (UPM), Serdang 43400, Selangor, Malaysia; dwrong753@gmail.com; 2 Laboratory of Food Safety and Food Integrity, Institute of Tropical Agriculture and Food Security, Universiti Putra Malaysia (UPM), Serdang 43400, Selangor, Malaysia; saranematbakhsh@gmail.com (S.N.); jinap@upm.edu.my (J.S.); noordiana@upm.edu.my (N.N.); 3 Department of Food Science, Faculty of Food Science and Technology, Universiti Putra Malaysia (UPM), Serdang 43400, Selangor, Malaysia; 4 School of Biosciences, Faculty of Health and Medical Sciences, Taylor’s University, Subang Jaya 47500, Selangor, Malaysia; peipei.chong@taylors.edu.my; 5 Department of Veterinary Preclinical Sciences, Faculty of Veterinary Medicine, Universiti Putra Malaysia (UPM), Serdang 43400, Selangor, Malaysia; hakim_idris@upm.edu.my; 6 Department of Biology, Faculty of Mathematic and Natural Science, Brawijaya University, JI. Veteran, Malang 65145, Indonesia; fatchiya@ub.ac.id

**Keywords:** chicken authentication, mislabeling, breed identification, omics, chemometrics

## Abstract

Chicken is known to be the most common meat type involved in food mislabeling and adulteration. Establishing a method to authenticate chicken content precisely and identifying chicken breeds as declared in processed food is crucial for protecting consumers’ rights. Categorizing the authentication method into their respective omics disciplines, such as genomics, transcriptomics, proteomics, lipidomics, metabolomics, and glycomics, and the implementation of bioinformatics or chemometrics in data analysis can assist the researcher in improving the currently available techniques. Designing a vast range of instruments and analytical methods at the molecular level is vital for overcoming the technical drawback in discriminating chicken from other species and even within its breed. This review aims to provide insight and highlight previous and current approaches suitable for countering different circumstances in chicken authentication.

## 1. Introduction

*Gallus gallus,* commonly known as a chicken, is a widely consumed species, and the demand for it keeps hiking up to 92.7 million metric tons in 2018 [1]. Due to the large-scale commercialization and easy accessibility of chicken meat and its related products, chicken has often been a common choice as a meat adulterant by food manufacturers to deliberately lower production costs [2]. Likewise, intentionally fraud consumers by substituting or mixing a cheaper breed of chicken into a highly valuable chicken breed is also practised as a marketing tactic by the food manufacturer to gain extra profit [3]. Moreover, raw and cooked chicken meat also exhibit allergenic effects in certain individuals [4]. Therefore, establishing a chicken authentication approach at a molecular level is urged in order to accurately verify label description and necessary information conforming to the chicken content in meat products.

Variation in chicken breeds genetic composition is one of the major factors determining growth rate, feed intake and feed efficiency in chicken farming [5,6,7]. Moreover, the chicken body parts such as abdominal fat pad and breast meat yields are also affected by genetic differences [8,9,10]. This raises the consumer interest in choosing chicken breeds according to their culinary and dietary preferences. Among various commercialized chicken strains, Cobb and Ross are known to be the strains most produced worldwide [11]. Meanwhile, several indigenous chicken breeds pose higher value than the commercialized broiler due to their unique texture, flavour, and taste and can be marketed as a geographical indication [12,13]. Food manufacturers often target these unique chicken strains as the subject of fraud [3]. This situation brings up the need for engagement in research and development for developing a rapid and reliable analytical approach for authenticating the integrity of chicken breeds, as claimed.

Subsequently, authenticating chicken in food can be achieved by identifying the presence of several stable and relevant biomarkers at the molecular level. In this sense, multi-omics discipline analytical approaches can facilitate the detection and identification of the target biomarkers. Omics studies include the entire flow of the central dogma of molecular biology, which starts from the information stored in DNA (genome) and is expressed (transcriptome) in the form of proteins (proteome), where the proteins play a role in anabolism and catabolism (metabolome) [14]. Beyond the product of each level of transition, there is a sort of biomolecule (DNA, proteins, metabolites, lipids, and carbohydrates) that can be used to discriminate chicken from other species and even variation among chicken subspecies.

Omics can be predominantly interpreted as a combination of diverse technologies applied for the qualification or quantification of a distinct molecular level. Unfortunately, it is impossible to detect a group of compounds by merely using a single approach. Hence, different omics and technologies should be developed and applied wisely in different circumstances to overcome each weakness found in a particular approach. Similarly to other analytical food authentication, chicken authentication based on omics involves indirect authentication of targeted and non-targeted analytical strategies. Targeted analysis refers to the detection and precise quantification of a particular set of secondary biological markers (e.g., DNA, RNA, proteins, glycans, metabolites, and lipids). The non-targeted analysis involves the simultaneous detection of up to hundreds of unspecified targets (mainly in metabolites and lipids), yielding a fingerprint reflecting the chicken and breeds identity. Chicken fingerprinting analysis mostly involves high-end analytical instruments such as mass spectrometry (MS) and its derivatives. On the other hand, fingerprints could also comprise data compiled from other analytical approaches or a combination of other complementary analytical methods [15].

Since chicken authentication covers a large scope of the discipline, this review aims to provide an overview of recent developments in omics-based analytical technologies to assess chicken in meat and meat products. This includes a brief discussion of the pros and cons of each approach, as well as remarkable innovation and application in overcoming issues faced in different food authentication circumstances.

## 2. Omics

The continuous development and improvement of novel and existing omics approaches have enabled the identification and quantification of biomolecules at high resolutions [16]. When these approaches combine together, it brings the origin of the authentication and quality assessment of food to the next level, invigorates food security, and protects consumer interest. Chicken meats are regarded as a food widely consumed by human beings as one of the main sources of dietary proteins. Hence, it is relevant to use the term “foodomics” introduced by Cifuentes [17] to describe the omics-related approach in chicken authentication. Foodomics can be defined as a scientific discipline combining food and nutrition studies via the incorporation of omics technologies to ameliorate consumers’ health, well-being, and knowledge [17]. Foodomics combines food and biological sciences such as genomics, transcriptomics, proteomics, glycomics, metabolomics, and its subdiscipline (lipidomics). Algorithms such as chemometrics or bioinformatics approaches were implemented to analyze large amounts of data yielded from omics. With the aid of statistical tools, the complex omics data can eventually be transformed into meaningful information and are able to interpret easily.

### 2.1. Genomics-Based Approaches

Genomics studies on the chicken are essential for exploring potential discriminative genetic markers to trace the chicken species and breeds in meat products. Genomics studies require the aids of bioinformatics tools to handle robust DNA sequence data extracted from the chicken genome. The emergence of next-generation sequencing (NGS) in this era facilitated DNA sequencing, replacing the previous gold standard―Sanger sequencing. Although NGS has significantly reduced the time required for DNA sequencing, NGS is still relatively costly at this time, and the penetration of this technology into the market will suppress the cost in its advent [18].

In the search for genetic markers for distinguishing three chicken breeds (White Leghorn, Korean domestic, and Araucana), Dongyep et al. [19] performed whole-genome re-sequencing on these domesticated chicken breeds using Illumina HiSeq2000. This study focuses on single nucleotide polymorphisms (SNPs) related to down/feather colour by aligning paired-end sequence reads to the chicken (galGal4) reference genomes. Another work by Bertolini et al. [20] used Ion Torrent NGS technology to identify chicken and other species in DNA mixture. Sequencing was performed on PCR products obtained from different couples of universal primers that amplify 12S and 16S rRNA mitochondrial DNA genes. Three different algorithms were used to assign the species based on sequence data. However, it was found that several primer pairs experienced limited efficiency of amplification and sequencing for avian species (chicken, pheasant, duck, goose, and pigeon) due to PCR competition. Overall, this benchtop technology has potential in species determination in meat products, producing massive reads in 3 to 4 h.

In addition, Cottenet et al. [21] recently reported the use of NGS on mitochondrial DNA fragments to identify meat species down to 1% (*w*/*w*). The sequences’ data were uploaded in SGS™ (Geneva, Switzerland). The All-Species ID software was aligned against a curated built-in database to identify the origin of the meat species. However, this NGS tool only allowed a rough quantification of the adulteration level. A similar Ion Torrent NGS was also performed by Ribani et al. [22] on 12S, and 16S mitochondrial rRNA genes were used to identify chicken and other meat species in highly processed, complex, and meat derived broths.

When it comes to chicken breed discrimination, different strategies can be applied. These included random amplified polymorphic DNA-PCR (RAPD-PCR), amplified fragment length polymorphism (AFLP), and PCR-RFLP in combination with bioinformatics analysis for breed discrimination. In brief, these approaches are associated with genomic fingerprints, and fingerprint differences between breeds are interpreted as genetic distances. RAPD is a technique that works by amplifying genomic DNA by using at least one short oligonucleotide primer in low stringency conditions resulting in multiple amplification products from loci distributed through the genome [23]. The RAPD method is simple but subject to low reproducibility. Conversely, microsatellite analysis is more reliable to this extent. Microsatellites are highly polymorphic and occur in all chromosomal regions, with up to several dozen alleles at each locus; hence, they can be easily scored using PCR [24]. The comparative analysis of RAPD and microsatellite polymorphism on chicken populations genetic diversity performed by Zhang et al. [25] concluded that microsatellite analysis could generate more accurate population relationship clustering on closely related populations than compared to RAPD.

AFLP is another PCR-based DNA fingerprinting method adapted from the RFLP and RAPD approaches [26]. AFLP offers advantages such as being highly abundant, highly polymorphic, locus-specific, easy to analyze, and does not require species-specific primers; therefore, it is suitable for population genetic studies [27]. This technique involves the ligation of adaptor molecules relative to restriction enzyme fragments, which subsequently serve as primer binding sites for PCR amplification [28]. Therefore, different sets of restriction enzymes or different primer pair combinations can yield large amounts of different AFLP fingerprints without prior sequence knowledge. For instance, Yu Shi et al. [29] and Gao et al. [30] performed a phylogenetic and genetic variation study using AFLP on 12 indigenous Chinese chicken breeds and one foreign breed. This study provides precious information regarding the genetic diversity, genetic relationships, and identification of chicken breeds in China. Another study from Fumiere et al. [13] successfully identified two strains specific markers based on two possible restriction enzyme combinations, *EcoR*I/*Mse*I or *EcoR*I/*Taq*I, to differentiate slow-growing from fast-growing chicken strains.

DNA-barcoding is another approach for chicken species and breeds identification. DNA barcoding can be carried out in the form of conventional full-length mitochondrial cytochrome *c* subunit I gene (≈650 bp) [31], but it may be challenged in highly processed food [32]. Instead, mini-barcode (DNA fragments within the full-length COI barcode region (≥100 bp)) can be alternatively used for species identification of processed food containing degraded DNA [33]. In this regard, Xing et al. [34] compared the efficiency of full- and mini-length DNA barcoding in animal-derived food in China. The COI barcodes of the samples are first amplified using PCR followed by bidirectional DNA sequencing. The barcode sequences were then compared to the reference libraries for species identification. The result showed the mini-barcode offered a useful alternative to the full-length barcode for species identification in terms of DNA degraded meat samples. However, it was found that detecting mixtures of DNA from multiple animal species is hard to achieve using this technique. For chicken breed discrimination, Peng et al. [35] had performed DNA barcoding on chicken mitochondrial COI to assess one new native chicken breed when identification based on morphological examination was difficult. Another five chicken breeds were compared to evaluate the efficacy of DNA barcoding. The study revealed that this approach offers advantages such as convenience, low cost, and rapid and pleasing accuracy in identifying different chicken breeds.

### 2.2. Classical DNA-Based Techniques

**Figure 1:**
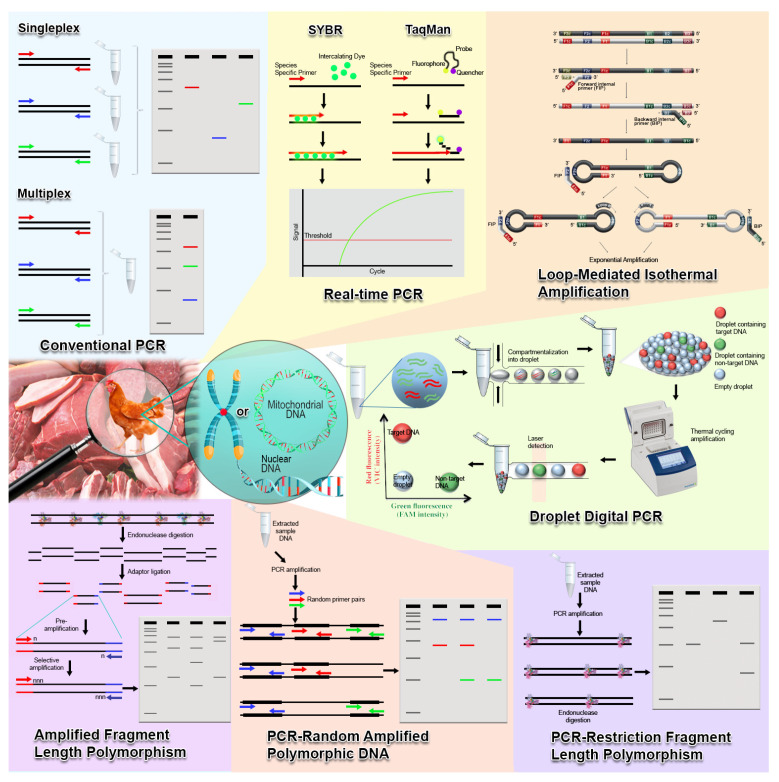
An infographic overview for classical DNA-based molecular techniques in chicken meat adulteration detection, species, and breeds identification. Conventional PCR can be performed to identify a species at a time (singleplex) or multiple species simultaneously (multiplex) using species-specific primer targeting either nuclear or mitochondrial DNA. Real-time PCR plays the same concept as conventional PCR but incorporating dyes to monitor the PCR product in real-time. Loop-mediated isothermal amplification amplifies the target species using a set of four to six specially designed primers under isothermal conditions. Droplet Digital PCR allows the detection of a very low number of targets in DNA mixture by fractionating samples into 20,000 droplets, followed by target amplification of target in each droplet and detection using a laser. PCR-RFLP amplifies a conserved DNA region followed by digestion of the PCR products using one or more restriction endonucleases. Restriction profile can be obtained from the variation in band formation from agarose gel electrophoresis. PCR-Random Amplified Polymorphic DNA amplifies random segments of DNA by PCR using a single arbitrary primer binds to different loci in different species; variation in band patterns can be used to discriminate species. Amplified fragment length polymorphism technique discriminates individual species or breeds based on the selective PCR amplification of restriction fragments caused by single nucleotide polymorphism from a total digest of genomic DNA.

Genetic traceability describes the use of DNA as a component to authenticate the species origin of an organism and its products. According to the European Parliament Resolution of 14 January 2014, DNA testing is suggested as a standard procedure for animal species determination for the purpose of fraud detection and control [36]. Hence, DNA-based techniques are placed in a pivotal position in authenticating the genuineness of animal products. Since DNA is ubiquitous and identically found inside almost every cell of the organism, DNA extraction can be performed on biological samples such as muscle, blood, and many other tissues in suspicious meat product specimens, followed by species identification based on molecular markers such as a nuclear or mitochondrial gene. DNA also offers higher stability than proteins in terms of heat processing. Although it can be fragmented by extensive heating, advanced DNA-based approaches still managed to identify DNA from different species available in a given specimen [37]. In addition to the genomics approaches, DNA-based techniques offer more directed identification of chicken meats origin by targeting genetic markers. The overview of the classical DNA-based techniques in the authentication of chicken species and breeds is summarized in Figure 1. Moreover, studies and their respective performance are systematically listed in Table 1.

In most cases, mitochondrial-based DNA analysis is preferred due to the high number of mitochondria that can be found in a cell, and many mitochondrial DNA copies stay inside each mitochondrion. Mitochondrial DNA is a potential candidate for species DNA identification given its maternal inheritance, low recombination probability, and the availability of conserved sequences. These reasons explain why mitochondrial DNA can serve as a naturally amplified source of genetic variation. In fact, most poultry DNA identification involves avian mitochondrial genes rather than nuclear DNA as a quantification component [45]. Despite the robustness of mitochondrial DNA in species identification assay, nuclear loci are more suitable for DNA quantification because of their diploidy, unlike multiple copy numbers of mitochondrial DNA [67]. Thus, it is relevant for targeting nuclear DNA for genotyping and quantification of samples that only require small amounts of template DNA. Moreover, multiplex amplification assays can be established based on multiple target sites within the nuclear genome for simultaneous species identification [68]. Table 2 summarizes the advantages and disadvantages of nuclear and mitochondrial DNA in muscle food origin identification.

Furthermore, the nucleotide sequence variations in mitochondrial genes such as 12S rRNA, 16S rRNA, cytochrome *b*, cytochrome *c* oxidase subunit (CO), D-loop, and NADH dehydrogenase (ND) (refer Table 2) are among the most prevalent genes used for discriminating chicken from other species in PCR assays. These molecular markers have shown good application in food adulteration inspection. PCR assays based on mitochondrial DNA are challenging for effectively discriminating closely related species (chicken from turkey) due to the high degree of sequence homology [44]. However, this challenge was overcome by using suitable markers such as the mitochondrial ND2 gene, as demonstrated by Kesmen et al. [44]. Moreover, mitochondrial DNA can be subjected to mutation at the primer binding region, resulting in erroneous detection of individual target species and different breeds [72]. Conversely, nuclear DNA is more conserved and stable for overcoming the issues mentioned earlier in species discrimination [62].

In DNA-based approaches for meat species identification, conventional PCR is a common technique used to amplify a small amount of contaminant DNA presence in meat samples but lacks quantitative capabilities, sensitivity, and is time-consuming, while many derivatives of it such as real time-PCR, multiplex-PCR, PCR-restriction fragment length polymorphism, and digital droplet-PCR offer much more advantages compared to conventional PCR. Notwithstanding, species identification by PCR in meat samples is challenged by the presence of inhibitory substances in meat sample matrices, and the risk of amplification inhibition is higher when more than 50 ng DNA is assayed [74]. Real-time PCR enables quantifying the amount of studied species DNA in a matrix by monitoring the increase in fluorescence signal [75]. This technique also offers advantages such as high sensitivity and specificity, wide detection range, and lower risk in carry-over contamination [76]. The high specificity of this assay is the result of the ability for intercalating fluorescent dyes such as SYBR Green to bind to all double-stranded DNA present, including any non-specific PCR products and the primer-dimer complex. Hence, it does not require additional electrophoresis steps such as conventional PCR in order to visualize the assay result. In addition, high resolution melting analysis (HRM) can be performed in real-time PCR to monitor the differences in melting temperature for differently originated species DNA. HRM can be applied in many analyses such as genotyping, mutation analysis, and methylation analysis but require the use of saturated dyes, HRM-capable real-time PCR device, and specialized bioinformatics software [77].

Additionally, PCR can be performed in a multiplex format using species-specific primers to detect different species simultaneously in one reaction, which saves cost and time. On the other hand, PCR-RFLP only offers species DNA fingerprinting based on banding patterns yielded from restriction endonuclease digestion of PCR amplicons, which is qualitative but not quantitative. Since the accurate quantification of a minute amount of target DNA in a matrix of non-interested DNA is highly challenged, a recently developed PCR technology known as digital droplet PCR (ddPCR) is able to encounter this difficulty [78]. ddPCR works by partitioning the PCR reaction mixture into tens of thousands of droplets, with each droplet harbouring an independent PCR reaction. In this sense, the target DNA copy number is determined based on the number of droplets positive for amplification of the target DNA. Hence, this approach allows absolute DNA quantification, does not involve standard curves as in real-time PCR, and enables high accuracy quantification of low concentration target DNA in a high background of non-interested DNA [79]. Furthermore, PCR-FINS is another DNA amplification method involving nucleotide sequencing and analysis using the NCBI basic local Alignment search tool (BLAST). This approach depends on the accurate selection of DNA regions and reference species [80].

Interestingly, nucleic acid sensor assay can also design based on PCR amplification to rapidly detect chicken adulteration. Xiao et al. [63] developed a chicken-specific PCR combined with a nucleic acid sensor test (Chicken-PCR-Sensor). This assay priorly amplifies the DNA extracted from meat specimen using chicken-specific primer, followed by visual detection of the amplicons using lateral flow antibody sensor assay. This novel approach shows a remarkable outcome with the ability to detect 0.01% adulterated chicken in the meat mixture within 2 to 3 min. Besides PCR-based DNA analysis, loop-mediated isothermal amplification (LAMP) is another alternative capable of amplifying target DNA at a constant temperature [81]. Unlike PCR, LAMP employs a different DNA polymerase and four to six different primers (two for PCR) to recognize six to eight distinct target gene sequences with high specificity and sensitivity [82]. Sul et al. [83] successfully developed a direct LAMP assay targeting the mitochondrial 16S rRNA gene in order to detect chicken in processed meat products, with a very low detection limit (10 fg). An on-site inspection was possible by using a LAMP assay with direct amplification and a portable fluorescence device.

### 2.3. Transcriptomics-Based Approaches

In addition to substituting chicken into more valuable minced meat, the incidence of adulterating relatively cheaper chicken offal/internal organs into skeletal muscle meat is defined as another form of food fraud [84]. To protect consumer interest, the European legislation had established the Quantitative Ingredient Declaration (QUID), which removes offal (liver, heart, and kidney) from the meat category and emphasizes that the label must declare the constituent of non-muscle tissue in meat products [85].

Therefore, transcriptomics is suitable for identifying the presence of offal in minced meat by targeting the product of transcription. Micro RNAs (miRNAs) are a class of endogenous non-coding RNAs comprising about 21 nucleotides long. This biomolecule is abundant in number, small in size (stable), and tissue-specific [86]. Owing to the attributes of specific expression of miRNAs in a specific tissue [87], miRNAs, therefore, are chosen as a potential biomarker to differentiate chicken organs from each other.

In the recent past, Vishnuraj et al. [88] developed an innovative miRNA-based approach to detect the presence of liver, heart, and gizzard in chicken skeletal meat. This study performed deep sequencing on miRNAs obtained from the different chicken body parts. Screening is performed on the differently expressed miRNAs in each tissue studied to select potential markers specific to each tissue. Three miRNAs were selected as tissue-specific markers for the gizzard and two for the heart and liver.

The selected markers are further applied on field samples by qRT-PCR assay to validate feasibility. Fortunately, the result is satisfying, with high sensitivity of detection (limit of detection = 0.01 ng/µL). The study also proved that the stability of miRNAs was enough to withstand heat treatment and could be used as markers in heat-treated products as well. To summarise, the application of transcriptomics in identifying the adulteration of non-muscle tissue shows a promising end. This approach only involves the same equipment used in DNA techniques and offers highly reproducible and direct results without incorporating complicated statistical analysis. Further studies on the stability of the miRNAs are needed to reassure that this technique is still relevant for harsh processed chicken meat products.

### 2.4. Proteomics-Based Approaches

When it comes to proteins, it serves as the necessary nutrients for the human diet and explains why humans consume chicken. Incorporating proteomics in chicken authentication makes it possible to confront the drawbacks of the DNA-based approach, wherein extreme food processing conditions such as heat and chemical treatment can potentially result in DNA degradation yielding non-specific DNA fragments [89]. Chicken authentication involves two main areas of proteomics, which include protein identification and characterization and differential proteomics. Protein identification and characterization are often practised in the discovery phase to search for suitable markers for species or breed discrimination. This can be performed by selecting a class of protein present commonly in different species (e.g., Troponin I, Myosin light chain, myofibrillar, and sarcoplasmic proteins). On the other hand, differential proteomics is applied after separating target protein from other junk and differentiating the species based on the quantitative information of specific proteins (variation in the relative abundance of marker protein among species).

The discovery of versatile species-specific markers for species identification is the benchmark for food authentication. Proteomics analysis typically involves a series of separation steps followed by identifying the proteins through mass spectrometry. Preliminary separation of proteins based on their isoelectric point (p*I*) and molecular weight (Mw) can be conducted using techniques such as two-dimensional electrophoresis and OFFGEL fractionation, wherein a more detailed separation of peptides and amino acids based on mass-to-charge (*m*/*z*) ratio requires mass spectrometry instruments. In most cases, a bottom-up or peptide-based approach is applied for potential marker screening, where a class of proteins from different species is digested using a proteolytic enzyme known as trypsin. The enzymatic digestion reaction will yield a number of peptide fragments. The peptides will further be subjected to mass spectrometry analysis for protein identification purposes. The protein can be eventually identified by comparing the MS experimental data to the calculated mass values from the peptide sequence database such as MASCOT.

**Figure 2:**
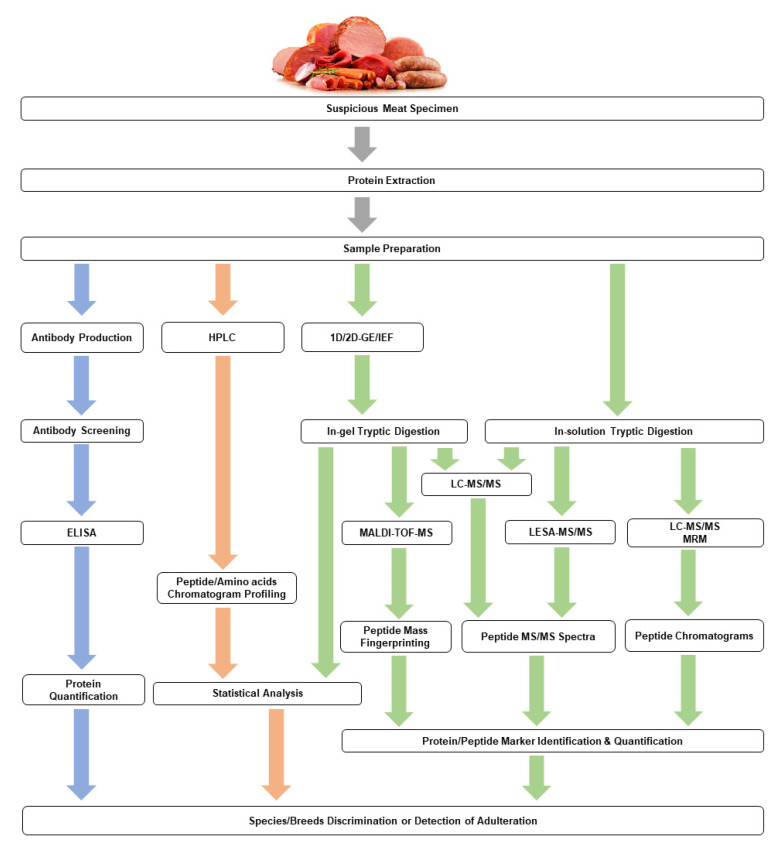
An overview for workflow performed previously in chicken proteome markers discovery. Blue arrows indicate the immunological approach, red arrows represent the chemical chromatogram profiling, and green arrows represent the bottom-up proteomics approach. The top-down proteomics approach is not shown due to no research reported using this approach in chicken authentication studies.

On the contrary, top-down approaches did not involve the enzymatic digestion of protein. Still, the protein is fragmented directly into peptides inside a mass spectrometer, and this approach is not commonly observed in food authentication due to the limitations in instrumentation advances [90]. Figure 2 presents the workflow of discovery of potential proteome markers for chicken authentication.

To confront the chicken meat substitution issue, Sentandreau et al. [91] developed a proteomics-based method by targeting a myofibrillar protein, myosin light chain 3. This method complies with the bottom-up approach but simultaneously consolidates the use of stable isotope-labelled peptides for quantification purposes. The sample peptides can be calculated by comparing the LC-ESI-MS/MS peak area values of the known initial amount of heavy peptide to the native peptide. This study also simplified the protein extraction/enrichment procedure by replacing the SDS-PAGE using OFFGEL fractionation followed by in-solution trypsin digestion. Another study from Montowska and Pospiech [92] also used electrophoretic mobility of myosin light chain isoform to differentiate among six species in minced meat products. This method is only applicable for mild processed meat and is not suitable for high salt content meat and meat heated over 100 °C. Furthermore, we need to note that the fermentation of raw meat such as salami sausages will result in the degradation of proteins by microbiological activities, thus increasing the challenges in species authentication on a protein basis. Most importantly, the information obtained from electrophoresis is only semi-quantitative. Therefore, not suitable for quantifying the level of adulteration.

In addition to electrophoretic techniques, ambient mass spectrometry is another method of choice for meat species authentication study due to detecting compounds directly from a biological surface at atmospheric pressure. Liquid extraction surface analysis mass spectrometry (LESA-MS) is an example of ambient MS, and the application of this instrument in the discovery of stable peptides for species authentication is demonstrated by Montowska et al. [93,94]. This technique works by dispensing an extraction solution on the sample’s surface to form a liquid/surface microjunction [95]. This approach exhibits high specificity in meat peptidomics studies and effectively analyzes digested or processed meat products.

Chou et al. [96] and Ramin Jorfi [97] had demonstrated the use of HPLC for species identification. These studies did not look up a specific proteome marker representing each species. Still, they used the proteome profile such as amino acid composition and chromatographic profile of electroactive peptides and amino acids. The Ramin Jorfi [97] study found that, with the aid of principal component analysis, amino acids profile generated using reversed-phase HPLC with pre-column derivatization can effectively differentiate chicken from other meats studied. Contrarily, although the chemical nature of major chromatographic peaks was not identified in Chou et al. [96] study, the developed HPLC with electrochemical detection method manages to detect meat adulteration as well as degradative changes in meat proteins.

Apart from advanced instrumentation, the immunological approach is also an alternative to meat speciation. Developing a biorecognition element detecting the species-specific meat antigen is crucial in this sense. For this purpose, Chen et al. [98] developed IgG class monoclonal antibodies to target a thermostable protein marker, troponin I. The developed antibody is then evaluated and implemented in an indirect ELISA. The developed immunoassay using the selected antibody shows good detectability down to 1% for all samples analyzed. Nevertheless, this assay was designed to detect animal origin in feedstuffs and, hence, not practically applicable to highly processed meat products due to the lack of data on the antigen thermostability.

Interestingly, it is possible to differentiate chicken breeds using the proteomics approach. The need to characterize the chicken breeds is demanded in food authentication and is also helpful for establishing a strategy for chicken population conservation. Zanetti et al. [99] had performed a proteomics study to characterize chicken breeds based on the differential expression of sarcoplasmic proteins. Similarly, De Liu et al. [100] also conducted a proteomic study on Korean native chicken and commercial broiler to understand the breed-specific differences in meat flavour on a molecular basis. These studies employ two-dimensional electrophoresis to separate the chicken skeletal muscle proteins and identify interested proteins spot using MALDI-TOF-MS. Subsequently, Likittrakulwong et al. [101] performed proteomic analysis on three chicken breeds for characterization purposes. This study analyzed chicken serum, which is odd compared to other studies that commonly use sarcoplasmic proteins. Therefore, the breed-specific markers identified in this study can be used for breeds conservation purposes but are not applicable in food forensics. To summarize, the application of proteomics approaches in chicken authentication is presented in Table 3.

### 2.5. Metabolomics-Based Approaches

Unlike other omics disciplines, metabolomics is a wide field comprising the identification and quantification of multiple metabolites produced from a biological system [107]. Metabolomics can be divided into two categories that include targeted and untargeted metabolomics [108]. Targeted metabolomics analysis works by extracting a chosen target molecule from a mixture of metabolites for identification and quantification purposes. This method is more relevant for species authentication and subsequently gives rise to a subdiscipline of metabolomics known as lipidomics. Untargeted metabolomics analyzes multiple metabolites with the aid of multivariate statistical analysis to reflect the changes in the metabolism status of a subject. Attributes of the types of metabolites in each organism are generally similar; thus, authenticating meat species based on untargeted metabolomics is rather challenging.

A novel metabolomics approach for meat species identification is reported by Zhou et al. [109]. This approach utilizes laser-ablation electrospray mass spectrometry (LAESI-MS) to directly analyze the biochemical information of meat samples without requiring any chemical pretreatment. A laser priorly ablates the meat specimen to generate particulates, followed by ionization by electrospray ionization and mass spectrometer identification to characterize the metabolites present in each meat species. When in use with the established PCA and PLS-DA model, the prediction accuracy of 100% for each meat species was achieved. Further variable importance in the projection values (VIP values) analysis, 18 markers for chicken meat identification, and 19, 18, 17, and 15 markers for duck, pork, beef, and mutton, respectively, have been successfully selected for meat authentication. This study demonstrated the potentiality of LAESI-MS, which takes only 30 s to generate mass data, and meat speciation can be readily performed using the established PCA and PLS-DA models. Future studies on meat mixture from different species using this approach are anticipated and expected to be promising.

On the other hand, the emergence of nuclear magnetic resonance (NMR) spectroscopy as a pivotal instrument in metabolomics study is also a method of choice in addition to mass spectrometry [110]. Recently, Kim et al. [111] employed two-dimensional quantitative NMR (2D qNMR) spectroscopy to discriminate chicken breeds based on the metabolic variation. By complementing 1D ^1^H NMR to 2D qNMR, previous issues encountered in 1D ^1^H NMR, such as the lack of quantification ability and overlapping issues, can be addressed. Principal component analysis (PCA) and orthogonal partial least squares-discriminant analysis (OPLS-DA) are employed to study the metabolites’ differences. The study found that Korean native chicken strains contain higher amounts of flavour enhancing metabolites and lower amounts of free amino acids than the broiler. This study proves the potential of 1D ^1^H NMR complement with 2D qNMR in acquiring interactive and accurate information on chicken breeds metabolite differences, which outperformed traditional chromatographic analysis.

### 2.6. Lipidomics-Based Approaches

**Figure 3:**
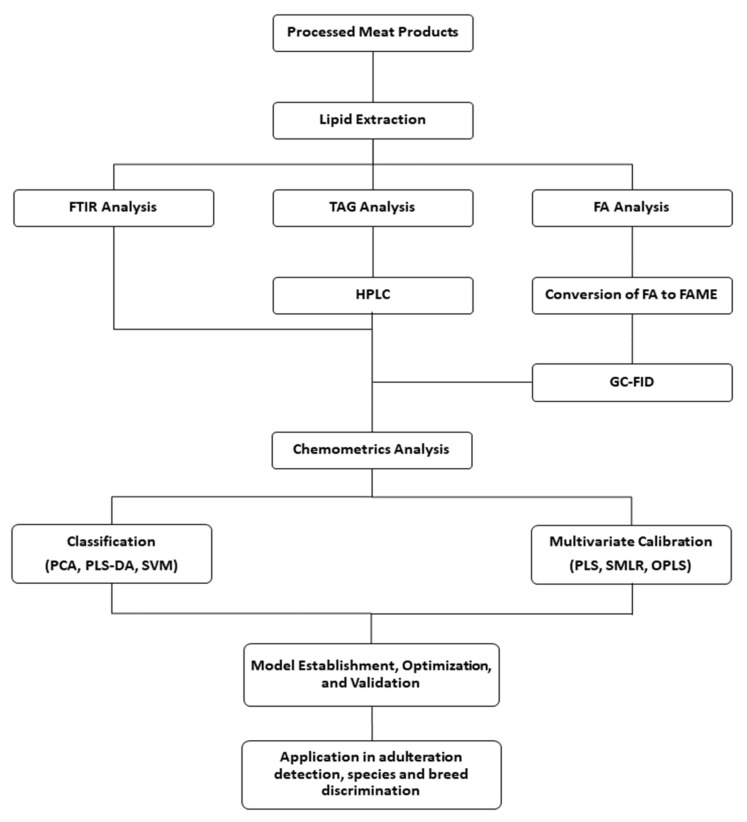
General lipidomic workflow in meat products analysis. TAG, triacylglycerol; FA, fatty acid; FAME, fatty acid methyl ester; GC-FID, gas chromatography with flame ionization detector.

In the past 20 years, lipidomics emerged as a subdiscipline of metabolomics together with the enhancement in analytical instruments and chemometrics [112]. Data generated from lipidomics analysis are often multivariate. Hence, principal component analysis (PCA) minimizes the data dimension by drawing out redundant information in all samples. The remaining data known as principal components can provide information about the maximum variation among different classes. On the other hand, partial least squares (PLS), partial least squares discriminant analysis (PLS-DA), and orthogonal partial least square discriminant analysis (OPLS-DA) are suitable for classifying or discriminating different groups of lipidomic data [113]. The general workflow of lipidomics in combination with chemometrics is illustrated in Figure 3, while Table 4 summarizes the studies of chicken authentication based on lipidomic approaches.

Chicken fat may serve as a potential adulterant in canola oil [114], butter [118], and also cod liver oil [116], owing to its highly identical fatty acid profiles and low cost. The presence of chicken fat in vegetable oil (canola oil) will infringe the interests of vegetarianism practitioners, whereas the mixing in other animal oil is known to be food fraud to consumers. Most of the food lipidomics studies aim to detect lard adulteration to defend the religious faiths of Islam, Judaism, and Hinduism followers [126].

Regarding the instrumentation in studying chicken lipidomes, FTIR is the most popular instrument due to its ability as a fingerprint analytical technique. FTIR offers an advantage in detecting closely similar fatty acids profiles from two different species that surpass the GC [116]. Generally, most studies encountered a common issue in discriminating fatty acid and triacylglyceride profiles of chicken fat from lard. This is mainly due to both mentioned species sharing a highly identical lipid profile. For instance, the use of cooling and heating thermograms from differential scanning calorimetry (DSC) by Marikkar [114] had failed to overcome this issue. Another study by Nizar et al. [117] tried coping with this issue by subjecting fatty acid distributional data obtained from GC-MS to PCA and successfully obtained stearic, oleic, and linoleic acids as parameters in differentiating chicken fats from lard. Accordingly, Saputra et al. [123] demonstrated the importance of applying a scatterplot screener program to process the undistinguishable pig and chicken fat spectrum wavelengths to select an appropriate fingerprinting wavelength region from FTIR spectra to classify all the fats precisely into corresponded species sources.

Other than FTIR-based approaches, the application of elemental analyzer-isotope ratio mass spectrometry (EA-IRMS) in the study of fatty acids, monoacylglycerol, and diacylglycerol (MAG and DAG) also shows remarkable performance in food authentication. The significant differences in carbon isotope ratio (δ ^13^C) values of fatty acids [117] and MAG and DAG [119] in animal fats show promising potential to be used for the discrimination of animal fats from different species. Likewise, incorporation of PCA is needed to classify the subject into the respective source of origin accurately.

Furthermore, near-infrared (NIR) spectroscopy is also demonstrated by Alfar et al. [120] to authenticate fats originating from beef, chicken, and lard. The NIR works by exciting a single covalent molecular bond at a 700–2500 nm region, as the molecular bonds are distinctive for different substances. The intensity and peak location of the NIR spectrum can provide information about the analyte’s chemical structure but does not reveal the concentration of each compound present in the analyte. Since NIR cannot profile the animal fats according to fatty acids composition, it detects the functional groups of these compounds. Due to this reason, this method requires extra care in handling the samples at a constant temperature, wherein the change in extraction temperature will result in changes in levels of spectrum absorbance or intensity. Unfortunately, although NIR is used with a support vector machine (SVM), the results indicate that this method can only classify lard from chicken and beef fat with 100% accuracy but failed to classify beef and chicken fats samples.

Mi et al. [121] reported comprehensive lipidomics studies on Taihe black-boned silky fowl (*Gallus gallus domesticus Brisson*) by profiling the lipid composition of this chicken from different ages, genders, and different body parts. Furthermore, a lipidomics comparison was made on the Taihe black-boned silky fowl with crossbred black-boned silky fowl. The result indicates that it is feasible to distinguish Taihe from crossbred black-boned silky fowls based on 47 statistically significant lipid molecules using OPLS-DA and ANOVA analysis. This finding provides insight into the possibility of chicken breeds authentication based on the lipidomics approach.

Recently, Hrbek et al. [124] developed a rapid screening method of meat TAGs profile using direct analysis in real-time coupled with high-resolution mass spectrometry (DART–HRMS). This method offers high-throughput, quick, and straightforward sample preparation and short scanning time compared to conventional PCR analysis. Conversely, DART–HRMS instrumentation is costly and only suitable for mass sample screening to select suspicious specimens even when chemometrics is applied for data classification. The author has yet to declare the necessity of DNA analysis for validating the adulteration event with respect to further suspicion.

### 2.7. Glycomics-Based Approaches

The application of glycomics in meat authentication has just emerged during these five years [127]. Shi et al. [128] first reported the use of protein glycosylation to qualitatively and quantitatively analyze five meat species, including chicken. This study involves the enzymatic pretreatment of a meat sample using the PNGase F enzyme to yield *N*-glycans. Hydrophilic interaction ultra-performance liquid chromatography (HILIC-UPLC) is preferred as the separation technique for *N*-Glycan labelling and analysis to overcome no retention on the typical reverse phase as glycans are highly polar molecules. Targeted sample peaks are then analyzed using matrix-assisted laser desorption/ionization time-of-flight mass spectrometry (MALDI-TOF MS), separating the glycans according to the molecular masses. A tandem mass spectrometry MS/MS is further carried out to enable the identification of *N*-glycans structure. In this case, principal component analysis (PCA) was employed to discriminate samples, whereas partial least squares (PLS) regression analysis was used to evaluate the adulteration ratio. Among the meat samples, the *N*-glycans profile exhibits the most significant number of peaks. What is noteworthy is that the poultry meat sample (chicken and duck) contains relatively more minor *N*-glycans structures than other mammal species. This study also successfully discriminated against mixed binary and ternary species samples quantitatively.

The study above described the significant compositional variation in *N*-glycans between different species as a profile to authenticate chicken or other species. The genes encoding the glycoenzymes participating in the synthesis of *N*-glycans are responsible for this structural specificity. Several advantages can be concluded from the use of the glycomics approach in chicken or species authentication. The use of *N*-glycan profiles of meats can overcome the drawbacks of the genomics approach in which the harsh processing of meat (e.g., roasting, frying, microwaving, boiling, and steaming) may potentially degrade DNA. Data processing in the glycomics approach is also much more straightforward than the spectroscopic approach, which is rapid and requires minimal sample preparation. The author also claimed that the proposed approach could be accomplished within four hours, which is generally considered prompt.

Regarding the downside of the Shi et al. [128] approach, the limit-of-detection (LOD) level for the studied samples showed only 2.2%. Thus, although the value is low enough to disclose adulteration, a lower value is preferred for detecting meat contamination. What needs reminding is that glycosylation is also related to an individuals’ health status and disease progression [129]. Hence, future studies must also verify whether the *N*-glycan profile differs significantly among chickens with different ages, health status, and chicken breeds.

## 3. Conclusions and Future Perspective

Chicken and its breed authentication are becoming more critical as the global consumer demand for chicken and its related meat products keeps increasing. Moreover, the growth in consumer awareness towards the meat product packaging information genuineness also invokes muscle food traceability and security. The detection of chicken adulteration based on molecular approaches is highly credentialed, but it requires complicated sample extraction and pretreatment methods. The genomics-based approach has emerged together with Next-Generation Sequencing technologies in species origin identification for meat products. Although NGS is fast and becoming less costly in meat species authentication, these tools are only useful in identifying hypothesis-free species in a given sample. In terms of DNA-based approaches, since many potential genetic markers have been identified in previous research, future works can focus on incorporating DNA markers into the development of DNA hybridization type biosensor as performed by Skouridou et al. [130]. Furthermore, developing a rapid on-site detection kit based on direct LAMP is also feasible owing to the feature that the assay can be performed at a constant temperature. However, the direct LAMP approach requires extra effort in primer design and a lack of multiplex capability as in PCR.

With the emergence in the transcriptomics discipline, the detection of chicken offal adulteration based on miRNA also precedes the genomics approach, which cannot achieve the same results. In proteomics, the advancement in mass spectrometry technologies has brought convenience to high-resolution peptide marker discovery. Moreover, the selected peptide markers correspond to the species that can be used in adulterant detection. Still, enzymatic pre-digestion of the meat protein using trypsin is inevitable for bottom-up proteomics, while only a more advanced mass spectrometer can bypass this step. From the author’s point of view, the discovered potential peptide marker can serve as a novel developed assay target in which biosensors can be designed according to the peptide marker wherein the protein matrix should be digested by the enzyme first. The discrimination of chicken breeds based on metabolomics can be achieved well using chromatographic-mass spectrometry and NMR spectroscopy. Future applications of these approaches in differentiating chicken from other species are anticipated. The non-targeted lipidomics studies use spectroscopic instruments, especially FTIR, which discriminate species based on functional group profiles. Recent attempts on chicken lipidomics study using LC-MS enable the discrimination of chicken breeds based on identifying lipid molecules profiles in detail and are potentially preferred as an approach for high-resolution lipidomics study.

Regarding glycomics, more effort should be placed on validating species-specific glycans by involving a higher number of samples and different health statuses and ages of given species. Since we are at the edge of Industrial Revolution 4.0, the application of big data tools for data mining and interpretation will be more easily accessible, thus, aiding the leap in chicken authentication. Using the robust data obtained from the previous experiments, linking the knowledge from different omics approaches is essential for inventing the novel, portable, miniaturized, and automated sensing technologies. Rapid and convenient muscle food assessment that simultaneously enhance the accuracy and resolution of currently available technologies will be the future trends in this research area.

## Figures and Tables

**Table 1:** Summary of classical DNA-based techniques in the application of detection and authentication of chicken in meat and meat products. Type of samples and performance (limit of detection and discriminating accuracy) are included when available.

Species/Breeds Involved	Main Technique	Main Markers	References	Detection Performance
Bovine, porcine, and chicken	qPCR	Species-specific SINEs	[38]	Limit of detection: 5 pg
Beef, pork, lamb, goat, chicken, turkey, and duck	qPCR	Nuclear IL-2 precursor gene	[39]	Detection level: 0.1%
Bovine, sheep, pig, and chicken	PCR	Mitochondrial 16S rRNA gene	[40]	Detection level: 0.1%
Beef, pork, horse, mutton, chicken, and turkey	qPCR	Mitochondrial cyt*b* gene	[41]	Detection level: 0.01%
Chicken, duck, pigeon, and pig	PCR	Mitochondrial D-loop gene	[42]	NA
Turkey, chicken, beef, pork, and sheep	qPCR	Mitochondrial 16S rRNA and cyt*b* genes	[43]	Detection level: 1%
Turkey, chicken, bovine, ovine, donkey, pork, and horse	qPCR	Mitochondrial ND2 gene.	[44]	Detection level: 0.001%
Chicken, duck, and turkey	qPCR	Nuclear TF-GB3 gene	[45]	Limit of detection: 5–50 pg
Pork, beef, chicken, and mutton	Multiplex-PCR	Mitochondrial COI gene	[46]	Detection level: 0.001 ng
Duck, partridge, pheasant, quail, chicken, and turkey	PCR	Mitochondrial cyt*b* gene	[47]	Detection level: 0.01% (*w*/*w*)
Processed chicken, bovine, and pork meats	PCR	Mitochondrial cyt*b* gene	[48]	Limit of detection: 1%
Processed beef meat products	PCR	Mitochondrial cyt*b* gene	[49]	Limit of detection: 0.5%
Beef, pork, chicken, rabbit, horse, and mutton	qPCR	Mitochondrial COI gene	[50]	Limit of detection: 0.1%
Bovine, porcine, chicken, and turkey	ddPCR	Mitochondrial cyt*b* gene	[51]	Limit of detection: 0.01–1.0% (*wt*/*wt*)
Pork, beef, horse, duck, ostrich, and chicken	Multiplex-qPCR	Mitochondrial cyt*b*, COI, and 16S rRNA genes	[52]	Detection level: 0.32 ng
Pork, beef, horse, rabbit, donkey, sheep, goat, dog, chicken, duck, pigeon, goose, and turkey	ddPCR	Nuclear RPA1 gene	[53]	Limit of detection: 0.1% (*w*/*w*)
Beef, sheep, pig, horse, rabbit, chicken, turkey, and quail	qPCR, HRM	Mitochondrial cyt*b* gene	[54]	Limit of detection: 0.1 ng
Chicken, pheasant, quail, Silky Fowl, pigs, cows, sheep, duck, goose, dog, rabbit, yak, horse, donkey, and fish	qPCR, Southern blot, and digital PCR	Nuclear Act*b* gene	[55]	Limit of detection: 10 pg
Processed meat products from 24 species, including chicken	LAMP	Mitochondrial 12S rRNA gene	[56]	Limit of detection: 10 fg
Beef, buffalo, chicken, cat, dog, pork, and fish	Heptaplex-PCR	Mitochondrial cyt*b*, ND5, and 16s rRNA genes.	[57]	Limit of detection: 0.01−0.001 ng
Processed meat products from pork, beef, and chicken	qPCR	NA	[58]	Limit of detection: 0.1% for beef and pork; 0.2% for chicken
Beef, donkey, chicken, and human	PCR	Mitochondrial 12S rRNA gene	[59]	NA
Pork, chicken, and beef	Multiplex-qPCR	Mitochondrial cyt*b* gene	[60]	Limit of detection: 0.1 pg
Beef, sheep, pork, goat, horse, chicken, rabbit, and turkey	PCR	Beta-tubulin intron III gene	[61]	Detection level: 0.5% and 1%
Sheep/goat, bovine, chicken, duck, and pig	Multiplex-PCR	Nuclear DNA	[62]	Limit of detection: 0.5 ng
Chicken, beef, mutton, pork, duck, goose, venison, horse meat, donkey meat, fish, shrimp, and crab	PCR-sensor	Mitochondrial cyt*b* gene	[63]	Detection level: 0.01%
Cattle, buffalo, goat, sheep, pig, and chicken	PCR-FINS	Mitochondrial cyt*b* gene and the ATP synthase F0 Subunit 8 genes	[64]	NA
Duck, chicken, goose, wild goose, quail, goat, sheep, pork, beef, horse, and donkey	Hexaplex-qPCR	Mitochondrial ND4, COI, COII, 12S rRNA, and 16S rRNA genes	[65]	Limit of detection: 0.01–0.1 ng
Chicken, mutton, beef, and pork	Multiplex-qPCR	Nuclear TGFB3, PRLR, ND5, and ACTB genes	[66]	Detection level: 0.002 ng

PCR, polymerase chain reaction; qPCR, real-time/quantitative polymerase chain reaction; SINEs, short interspersed elements; IL-2, interleukin-2; cyt*b*, cytochrome *b*; ND2, NADH dehydrogenase subunit 2; COI, cytochrome *c* oxidase subunit I; ddPCR, droplet digital polymerase chain reaction; RPA1, replication protein A1; HRM, high resolution melting analysis; LAMP, direct loop-mediated isothermal amplification assay; ND5, NADH dehydrogenase 5; PCR-FINS, polymerase chain reaction-forensically informative sequencing; TGFB3, transforming growth factor beta-3; PRLR, prolactin receptor; ACTB, beta-actin.

**Table 2:** A summary of advantages and disadvantages of nuclear and mitochondrial DNA in muscle food origin identification.

DNA	Advantages	Disadvantages
Nuclear	Sequence information is conserved and stable [62].	More susceptible to fragmentation in extensive food processing than mitochondrial DNA [70].
	Diploidy (suitable for genotyping) [68].	
	Multiplex species identification at multiple target sites [68].	
	Enable accurate quantification of meat weight based on the DNA copy number [69].	
	Contains repetitive sequences (e.g., short interspersed nuclear elements (SINE) and long interspersed nuclear elements (LINE)) which can serve as amplification products, lowering the limit of detection [67].	
Mitochondrial	High copy number per cell (≈2500 copies) and varies in different tissues [71,72].	Subject to mutation at primer binding region [72].
	Higher probability of obtaining positive results in fragmented DNA caused by intense food processing [73].	
	Relatively higher in mutation rate than nuclear genes (suitable to discriminating closely related species, e.g., chicken vs turkey) [70].	Quantification of meat by transforming copy numbers to the weight proportion of meat is challenging [72].
	More resistant to fragmentation by heat compared to nuclear DNA [70].	

**Table 3:** Summary of proteomics application in chicken authentication. Type of samples and performance (limit of detection and discriminating accuracy) are included where available.

**Purpose of Analysis**	Main Technique	Statistical Analysis	Main Markers	References	Highlight
Detection of porcine, bovine, ovine, equine, deer, chicken, and turkey based on immunological approach.	ELISA	-	Troponin I (TnI)	[98]	A class of monoclonal antibodies against the thermostable troponin I marker was found to be able to recognize all of the meats. The detectability of the assay was less than 1% for all the species analyzed.
Differentiation of meat products from chicken and other 14 species based on electrochemical profiles.	HPLC-EC	-	Chromatogram peaks of electroactive peptides and amino acids.	[96]	The method involves simple extraction steps and may be applicable to fresh or cooked meats. Treatment of the meats at different harsh temperatures changed the intensity but not the pattern of species-specific peaks.
Preliminary proteomic study in 3 chicken breeds.	2D-GE, MALDI-TOF-MS	SAM	Breed-specific sarcoplasmic proteins.	[99]	Two categories of breeds-specific proteins were identified—breed-specific proteins and up or down expressed proteins in specific breeds.
Detection of chicken meat within mixed meat preparations.	OFFGEL-IEF, MALDI-TOF-MS, LC-MS/MS	-	Peptides from trypsin digestion of myosin light chain 3.	[91]	Two peptides were selected as chicken specific biomarkers; LC-ESI-MS/MS allows high sensitivity detection up to 0.5% *w*/*v* chicken meat presented in pork meat.
Differentiation of cattle, pig, chicken, turkey, duck, and goose based on differential expression of myosin light chain (MLC) isoforms.	2D-GE, MALDI-TOF-MS		Myosin light chain (MLC) isoforms.	[92]	MLC3f was selected as the most versatile marker possible to differentiate between the given five species.
Differentiation of pork from beef, mutton, chevon, and chicken based on their primary amino acid contents.	HPLC	PCA	Amino acids content.	[97]	Serine and histidine were identified as the main amino acids for differentiating chicken from the other meats studied, while serine, alanine, and valine could differentiate pork and chicken.
Identification of chicken breed-specific differences in terms of meat flavour between Korean native chickens and commercial broilers.	2D-GE, MALDI-TOF-MS	-	Skeletal muscle proteins.	[100]	Three proteins spots were found to increase in expression in Korean native chickens, while four proteins showed an increase in commercial broilers.
Searching of stable proteins differentiating cattle, pig, chicken, turkey, duck, and goose.	2D-GE, MALDI-TOF	-	Skeletal muscle proteins.	[102]	Significant differences in serum albumin, apolipoprotein B, HSP27, H-FABP, ATP synthase, cytochrome bc-1 subunit 1, and alpha-ETF can be considered to be used as markers in the authentication of meat products.
Selection and identification of heat-stable and species-specific peptide markers from beef, pork, horse, chicken, and turkey.	LESA-MS	PCA-X, OPLS-DA	Peptides from skeletal muscle proteins.	[94]	Nine chicken-specific peptides were identified. The limit of detection for chicken was 5% (*w*/*w*), and another two chicken peptides (not species-specific) were determined at 1% (*w*/*w*).
Authentication of processed beef, pork, horse, chicken, and turkey meat based on heat-stable peptide markers.	LESA-MS	-	Peptides from myofibrillar and sarcoplasmic proteins.	[93]	This study had identified six heat-stable chicken-specific peptide markers derived from myofibrillar and sarcoplasmic proteins.
Searching of protein markers for discrimination of beef, pork, chicken, and duck.	1D-GE, LC-MS/MS	-	Sarcoplasmic and myofibrillar proteins.	[103]	Four proteins were identified and able to discriminate mammals from poultry by differences in electrophoretic mobility; each species can be further identified through LC-MS/MS analysis.
To search for heat-stable peptide biomarkers in cooked meats of pork, chicken, duck, beef, and sheep.	UPLC-MS, MRM	-	Peptides from myofibrillar and sarcoplasmic proteins	[104]	After confirmation by the MRM method, six heat-stable chicken-specific peptides were found; three from six were novel.
Proteomic determination of three breeds of chickens.	LC-MS/MS	-	Peptides from serum proteins.	[101]	Two peptides were specific to Kai-Tor; one for commercial layer hen and one for white tail yellow chicken. A total of 12 proteins are found expressed differently in the three breeds.
Differentiation of duck, goose, and chicken inprocessed meat products based on the species-specific peptide.	LC-MS/MS	-	Peptides from skeletal muscle.	[105]	Ten chicken-specific peptides were monitored with high confidence using the qualitative LC-QQQ multiple reaction monitoring (MRM) method.
Authentication of chicken, duck, goose, guinea fowl, ostrich, pheasant, pigeon, quail, and turkey in raw and heated meat based on peptides marker.	HPLC-QTOF-MS/MS, LC-HRMS	-	Peptides from skeletal muscle.	[106]	Three chicken-specific peptides and one common turkey/chicken peptide were identified.

ELISA, enzyme-linked immunosorbent assay; HPLC-EC, high-performance liquid chromatography with electrochemical detection; SAM, significance analysis of microarrays, MALDI-TOF MS, matrix-assisted laser desorption ionization-time of flight mass spectrometry; PCA, principal component analysis; 2D-GE, two dimensional gel electrophoresis; IEF, isoelectric focusing; LESA-MS, liquid extraction surface analysis mass spectrometry; PCA-X, unsupervised principal component analysis; OPLS-DA, orthogonal partial least-squares discriminant analysis; PCA, principal component analysis; 1D-GE, one-dimensional gel electrophoresis; HPLC-MS/MS, high performance liquid chromatography-tandem mass spectrometry; MRM–MS, multiple reaction monitoring mass spectrometry; UPLC-MS, ultra-performance liquid chromatography-mass spectrometry; MRM, multiple reaction monitoring; HPLC-QTOF-MS/MS, high performance liquid chromatography-quadrupole-time of flight-tandem mass spectrometry; LC-HRMS, liquid chromatography–high-resolution mass spectrometry.

**Table 4:** Summary of lipidomic application in chicken authentication. Type of samples and performance (limit of detection and discriminating accuracy) are included when available.

**Purpose of Analysis**	Main Instrument	Statistical Analysis	Markers/Differentiation Features	References	Highlight
Analysis of tallow, lard, and chicken fat adulterations in canola oil.	DSC, HPLC, GC-FID	SMLR	Thermogram profile.	[114]	Chicken fat adulteration is impossible to be determined under DSC thermoprofiling.
Analysis of lard, body fats of lamb, cow, and chicken.	FTIR	PLS-DA	FTIR spectrum at fingerprint region (1500–900 cm^−1^) of lipid components.	[115]	The equation obtained from the calibration model can predict lard mixed with cow and chicken fat percentage at 1500–900 cm^−1^.
Analysis of cod liver oil, mutton fat, chicken fat, and beef fat.	FTIR	PLS-DA	FTIR mid-region (4000–650 cm^−1^).	[116]	PLS model can be used for the quantification of chickenfat in CLO with 100% accuracy.
Analysis of lard, chicken fat, beef fat, and mutton fat.	GC-MS, EA-IRMS	PCA	Stearic, oleic, and linoleic acids; carbon isotope ratios (δ ^13^C).	[117]	PCA of stearic, oleic, and linoleic acids data and significant differences in the values of carbon isotope ratios (δ ^13^C) of all animal fats can potentially discriminate meat species.
Analysis of chicken fat adulteration in butter	FTIR, GC-FID	PLS	FTIR spectrum at fingerprint region of (1200–1000 cm^−1^).	[118]	PLS can be successfully used to quantify the level of chicken fat adulterant with R^2^ of 0.981 at the selected fingerprint region of 1200–1000 cm^−1^.
Acylglycerols analysis of lard, chicken fat, beef fat, and mutton fat.	GC-MS, EA-IRMS	PCA	MAG and DAG profiles; carbon isotope ratios (δ ^13^C).	[119]	The presence of small amounts of arachidic acid and differences in the proportions of several fatty acids in the chicken diacylglycerols can differentiate chicken from lard. Variation in δ ^13^C values can also discriminate MAG and DAG in different species.
To authenticate fats originated from beef, chicken, and lard.	NIR	SVM	Wavelength region from 1300 to 2200 nm.	[120]	Using the developed SVM model, lard can be classified 100% correctly from chicken and beef fat, but only 86.67% accuracy was obtained when the three fats were classified together.
Lipid composition characterization of Taihe black-boned silky fowls and comparison to crossbred black-boned silky fowls.	UPLC/MS/MS, Q-TOF/MS	OPLS-DA	47 lipid molecules as markers to distinguish Taihe and crossbred black-boned silky fowls.	[121]	OPLS-DA analysis reveals 47 lipid compounds were statistically significant and can be used as potentialmarkers for the authentication of Taihe black-boned silky fowl.
Post-heat treated lard differentiation from chicken fats, mutton, tallow, and palm-based shortening.	FTIR	PCA, k-mean CA, LDA	Wavenumbers at region 3488–3980, 2160–2300, and 1200–1900 cm^−1^.	[122]	The combination of PCA with k-mean CA was able to differentiate heated fats according to their origin. LDA only possesses 80.5% classification accuracy where mutton and tallow cannot be classified correctly.
Wavelength profiling in a different mixture of fat samples containing chicken, lamb, beef, and palm oil.	FTIR	PCA	Wavelength at 1236 and 3007 cm^−1^.	[123]	The biomarker wavelengths identified from the spectra of the studied samples at positions 1236 and 3007 cm^−1^ separated at notable distances can be used to discriminate the fat from different species.
Triacylglycerols (TAGs) fingerprinting on beef, pork, chicken in meat products	DART–HRMS	PCA, PLS-DA	3 TAGs ion *m*/*z*.	[124]	DART–HRMS could be used primarily as a screening method, and suspected samples are required to be confirmed by PCR.
Profiling of lard with beef tallow, mutton tallow, and chicken fat.	GC-FID, HPLC, DSC	ANOVA, PCA	Score plot of 7 fatty acid composition, OOL/SPO ratio, and thermogram profile.	[125]	Score plot of PCA model, a significant difference in OOL/SPO ratio and thermal profile can provide a basis for differentiating chicken fat from lard.

SMLR, stepwise multiple linear regression analysis; DSC, differential scanning calorimetry; GC-FID, gas chromatography with flame ionization detector; FTIR, Fourier transform infrared spectroscopy; EA-IRMS, elemental analyzer–isotope ratio mass spectrometry; NIR, near-infrared spectroscopy; SVM, support vector machine; MAG, Monoacylglycerols; DAG, diacylglycerols; PLSR, partial least square regression; OPLS-DA, orthogonal partial least squares-discriminant analysis; UPLC/MS/MS, ultra-performance liquid chromatography-tandem mass spectrometry; k-mean CA, k-mean cluster analysis; LDA, linear discriminant analysis; DART–HRMS, direct analysis in real-time coupled with high-resolution mass spectrometry; PLS-DA, partial least squares discriminant analysis; OOL/SPO, oleic oleic linoleic/stearic palmitic oleic.

## Data Availability

Not applicable.
